# Two-phase equilibrium states in individual Cu–Ni nanoparticles: size, depletion and hysteresis effects

**DOI:** 10.3762/bjnano.6.185

**Published:** 2015-08-28

**Authors:** Aram S Shirinyan

**Affiliations:** 1 “Physicochemical materials science” center of National Academy of Sciences of Ukraine and Kyiv National University, Physics Faculty, Kyiv Taras Shevchenko National University, vul. Volodymyrska 61, 01601, Kyiv, Ukraine, phone: +380972984512, fax: +380445262326

**Keywords:** chemical depletion, nanomelting and nanosolidification loops, phase diagram of isolated nanoparticle, surface-induced size effect, thermodynamic approach

## Abstract

In isolated bimetallic nanoscale systems the limit amount of matter and surface-induced size effects can change the thermodynamics of first-order phase transformation. In this paper we present theoretical modification of Gibbs free energy concept describing first-order phase transformation of binary alloyed nanoparticles taking into account size effects as well as depletion and hysteresis effects. In such a way the hysteresis in a form of nonsymmetry for forth and back transforming paths takes place; compositional splitting and the loops-like splitted path on the size dependent temperature–composition phase diagram occur. Our calculations for individual Cu–Ni nanoparticle show that one must differentiate the solubility curves and the equilibrium loops (discussed here in term of solidification and melting loops). For the first time we have calculated and present here on the temperature–composition phase diagram the nanomelting loop at the size of 80 nm and the nanosolidification loop at the size of 25 nm for an individual Cu–Ni nanoparticle. So we observe the difference between the size-dependent phase diagram and solubility diagram, between two-phase equilibrium curves and solubility curves; also intersection of nanoliquidus and nanosolidus is available. These findings lead to the necessity to reconsider such basic concepts in materials science as phase diagram and solubility diagram.

## Introduction

One of the key questions in nanoscience is related to the problem of equilibrium phase diagrams variation for multicomponent finite systems with size decreasing. One of the most extensively studied size effects (first and foremost for pure materials) is the size-dependent melting temperature shift which is usually observed and explained in accordance to the so called capillary effect (surface-to-volume ratio or Laplace pressure) [1–3]. Somewhat less attention has been paid to binary and multicomponent nanosystems where the phase transition temperature becomes the function of composition as well [4–7]. The size and composition dependent results have been obtained mainly for melting and solidification of nanoparticles and they demonstrate the increase of solubilities of chemical elements, shift of equilibrium curves at phase diagrams downward in temperatures, etc. [8–13]. Hereby most of investigations have the restriction comparing only the energies of entire solid and entire liquid nanosystems. The problem is that the melting and solidification of nanomaterials are examples of the first-order phase transformations which start from a new phase nucleation (nucleation energy barrier) and it should be taken into account.

Size effects in multicomponent nanomaterials, where the first-order phase transformation starts from a nucleation and includes a change of composition of chemical elements, are accompanied with the less known “chemical depletion” effect: the amount of one of the chemical components may not be sufficient for the formation of a new phase nucleus of the different composition [11–13]. The similar arguments may be applied for the cases of density change during the nucleation in finite systems [14] and for grain boundary segregation problem as a successful approach to stabilize nanocrystalline materials against grain growth [15–17]. Chemical depletion is similar to oxygen starvation in medicine (also called as hypoxia) [18]. The origin of hypoxia is the same – chemical depletion by oxygen atoms when oxygen supply is insufficient. In recent papers devoted to the size and depletion effects the existence of compositional splitting and of the loops-like splitted path on the size dependent temperature–composition (*T–X*) phase diagram has been substantiated [19–21]. Unfortunately, many recently published works using the framework of thermodynamics do not take into account the chemical depletion factor. Moreover, classical Gibbs thermodynamics for nucleation does not consider the composition change in a matrix around the new formed nucleus supposing that such changes are neglectful. As we shall see further this is not true for closed nanoscale systems.

The present communication is directed to gaining the new knowledge of basic principles and specific features of materials stabilization processes related to the non-negligible size and depletion effects (based on calculations for individual Cu–Ni nanoparticles) providing new insights into first-order phase transition problem for nanosystems and basic concepts of phase diagram and solubility diagram in materials science.

The structure of the paper is as follows. First we give in brief the general thermodynamic approach explaining the influence of sizes and depletion on modification of Gibbs thermodynamics for multicomponent nanoscale systems. For simplicity we restrict the consideration by bimetallic individual nanoparticles. Next the *T–X* diagrams for an individual nanoparticle in solid–liquid two-phase region based upon the condition of the energy minimum are constructed and the difference between the equilibrium and solubility curves is explained. The concluding remarks are given in last part of the manuscript.

## Theory: thermodynamic approach for phase transformation

### Surface-induced size effect

Let us first briefly remember the size effect on the shift of phase diagram curves based on general thermodynamic approach. For a bulk material classical thermodynamics finds the equilibrium states related to the concavity (or convexity) of energy potentials after the so called Gibbs method of geometrical thermodynamics: first one has to plot the Gibbs free energy densities as functions of composition and then find the conditions for minimal energy of a given system by using the rule of common tangent [22].

Corresponding modification and theoretical descriptions for nanosystems may be done taking into account the additional surface energies of nanometric systems. Such approach explains, for example, the ‘anomalous’ appearance of metastable phases (from bulk point of view) in nanosized systems which are related to the change of the conditions for the phase equilibrium. Stable state exists when the system is in its lowest energy condition. As result the stable phase in bulk material (say, phase 1) is one which has the lowest bulk Gibbs free energy density *g* (energy per number of atoms in a system): *g*_1_ < *g*_2_. Subindexes 1 and 2 are referred to the phase 1 or phase 2, respectively. In a multicomponent system it also depends on such factors as molar fraction of a chemical element *X* (composition), temperature *T*, so that one can write:

[1]
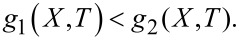


This condition has another form if one deals with a solution which is described by single Gibbs free energy density *g*_bulk_(*X*,*T*) dependence for both phases:

[2]
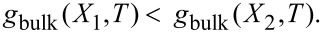


Here *X*_1_ and *X*_2_ are the molar fractions of a component in phase 1 and phase 2, respectively.

In a nanometric particle one has to take into consideration the surface and interface free energies, *σ*(*X*,*T*), which can dramatically change the equilibrium conditions [11]. Then Gibbs free energy density of a spherical nanoparticle having the total number of atoms *N*_0_ and the surface area can be defined as:

for phase 1 case –

[3]
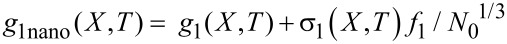


for phase 2 case –

[4]
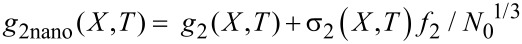


for solution model –

[5]
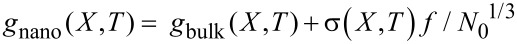


Here the *f*_1_, *f*_2_ and *f* are shape factors and the value *f*/*N*_0_^1/3^ represents the well-known surface-to-volume ratio; *σ*_1_(*X*,*T*) and *σ*_2_(*X*,*T*) are surface energy functions of phase 1 and phase 2, respectively. As one can see in Equations 3–5 to the origin of size effect belong the finite volume or number of atoms, the surface area and surface energy. One may observe also that the Gibbs free energy density in a nanoscale system is increased by the surface energy input.

**Figure 1:**
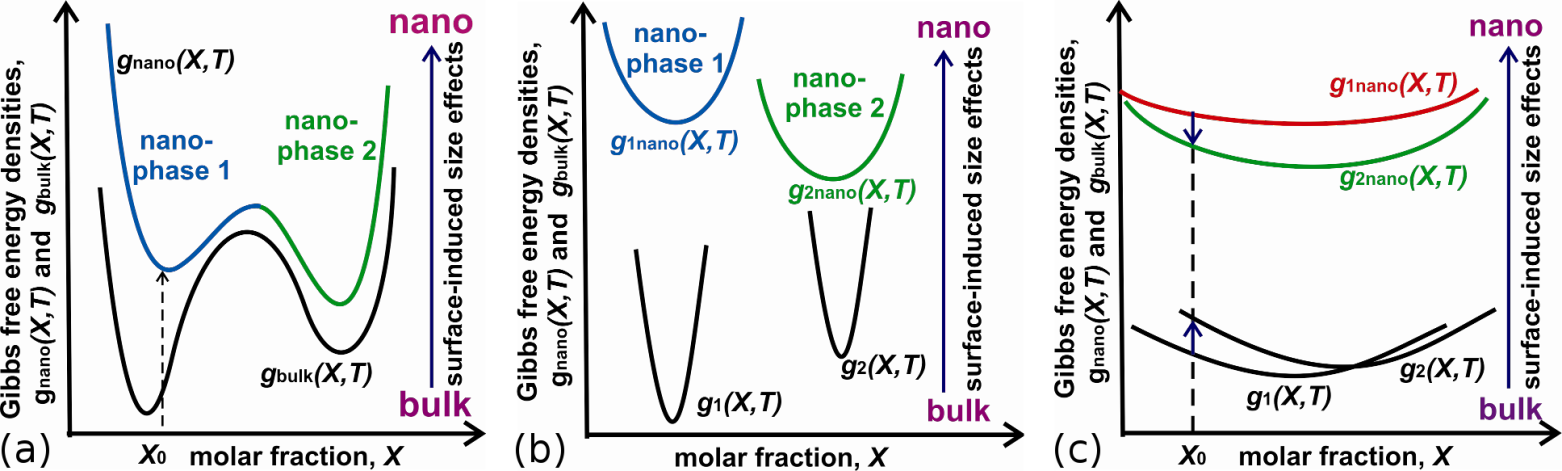
Surface-induced size effect on the shift of equilibrium states and solubility limits when the metastable in a bulk phase 2 becomes stable one in a nanometric volume: (a) – composition dependence of energy density for the solution case, (b) – case of parabolic dependences of Gibbs free energy densities of different phases, (c) – case of a isoconcentrational transformation. Black color curves *g*_bulk_(*X*,*T*), *g*_1_(*X*,*T*) and *g*_2_(*X*,*T*) characterize the energy density dependence on composition for given phases in a bulk case, color curves *g*_nano_(*X*,*T*)*, g*_1nano_(*X*,*T*) and *g*_2nano_(*X*,*T*) are shifted Gibbs free energy densities of given phases due to Laplace pressure. The value *X*_0_ is the initial composition. The solubilities of chemical elements are shifted as compared with bulk situation (if one uses the rule of common tangent).

If the nanophase 2 has the smaller surface free energy than the one for nanophase 1 σ_2_(*X*,*T*) *<* σ_1_(*X*,*T*), then so it may become the stable one (Figure 1) because of new conditions:

[6]
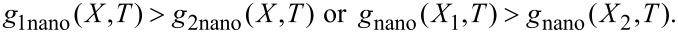


The critical number of atoms *N*_0_^*^ inside a particle transforming from nanophase 1 to nanophase 2 obeys the condition *g*_1nano_(*X*,*T*) = *g*_2nano_(*X*,*T*):

[7]
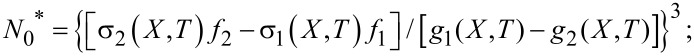


or the condition *g*_nano_(*X*_1_,*T*) = *g*_nano_(*X*_2_,*T*) for solution case:

[8]
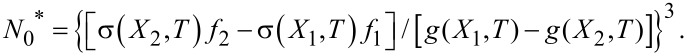


For a small volume of such a system (consisting the number of atoms *N*_0_) the nanophase 2 will be advantageous as compared with nanophase 1 when: *N*_0_ < *N*_0_^*^. The experimental examples of such kind surface-induced size effect have been found for different systems (mainly for pure metals and polymorphic transitions when bulk bcc structures transform to fcc or hcp types in a nanoscale) [23–24]. Figure 1 shows three qualitative situations concerning the effects of size and composition dependence of the surface energy on the first-order phase transformation in nanovolumes.

The first-order phase transformations are described as transformations following the nucleation of new phase clusters [22]. If the nucleation energy barrier ΔG* is very high (more than the about 50*kT*, *k* is the Boltzmann constant) then the phase transformation will be suppressed. That is why the nucleation must be taken into account in the thermodynamic calculations and it is critical for multicomponent nanosystems in terms of chemical depletion. In this respect the cases (a,b) in Figure 1 correspond to the formation of a new phase 2 nucleus with different composition and has additional constrains, as expected, due to depletion effect.

### Effect of chemical depletion on equilibrium states

**Figure 2:**
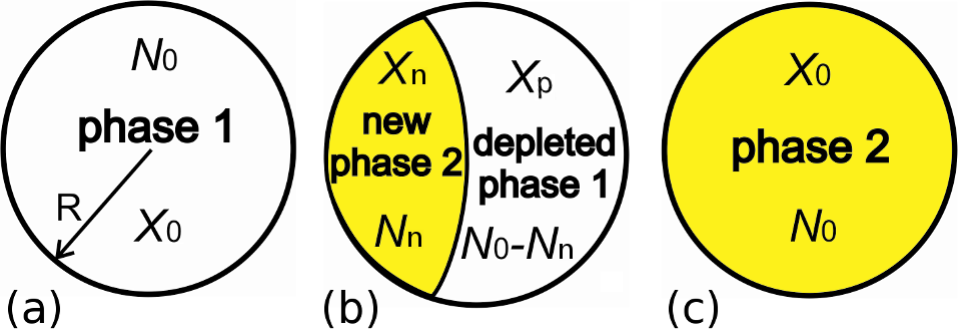
Possible phase transformation mode: an individual nanometric particle of initial composition *X*_0_ in single-phase state (a) and the same nanoparticle after new phase nucleation in two-phase configuration (b) and at the end of phase transformation (c). *R* – radius of spherical nanosized particle.

If a nucleus of a new phase 2 of composition *X*_n_ and number of atoms *N*_n_ are formed at the surface or inside an isolated nanoparticle (with initial composition *X*_0_ and number of atoms *N*_0_) then there exist the compositional changes *X*_0_ → *X*_p_ around the nucleus and the chemical depletion appears Δ*X = X*_0_ *– X*_p_ (*X*_p_ is the depleted composition around the nucleus, Figure 2). The minimal number of atoms in a nanosystem *N*_0_^**^ for the single new phase embryo formation may be found from the matter conservation law: *X*_0_·*N*_0_^**^ *= X*_n_·*N*_n_ and in such a way the value *N*_0_ for phase transition should be bigger than *N*_0_^**^:

[9]
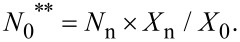


Both quantities *N*_0_^*^ and *N*_0_^**^ are important for multicomponent nanoparticles. That is why depletion dependent transformation in this work means the new nano-sized structure formation driven by both limit size and conservation of matter.

In general when new phase appears (Figure 2) the compositions *X*_n_, *X*_p_ and *X*_0_ obey lever rule equation:

[10]
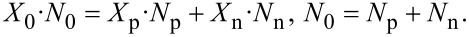


In our case (fixed pressure and temperature) the thermodynamic potential will be the Gibbs free energy of a nanosystem being the sum of two parts: the bulk and the surface contributions. For chosen phase transition mode from single-phase state to two-phase morphology (Figure 2a,b) the Gibbs free energy change Δ*G*(*X*_n_,*N*_n_,*T*) has the form:

[11]
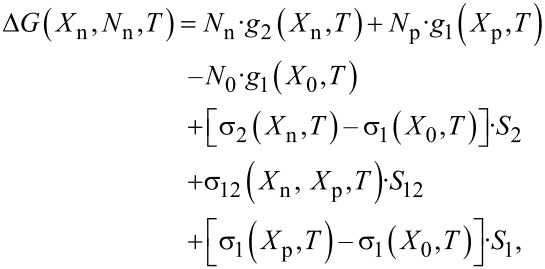


In Equation 11 the quantities *S*_1_ is the external surface area of phase 1, *S*_2_ is the external surface area of phase 2, *S*_12_ is the interphase area, σ_12_(*X*_n_,*X*_p_,*T*) is interphase energy being the function of both compositions *X*_n_, *X*_p_ and the temperature. That expression represents the modification of Gibbs thermodynamics for muticomponent nanosystems and differs from commonly known and used expression of classic nucleation theory for Gibbs free energy change. The main difference is in the second term *N*_p_·*g*_1_(*X*_p_,*T*) which is outgoing in a bulk and accounts the chemical depletion effect in a nano. As result it changes the thermodynamics of phase transformation as compared with classical Gibbs theory.

**Figure 3:**
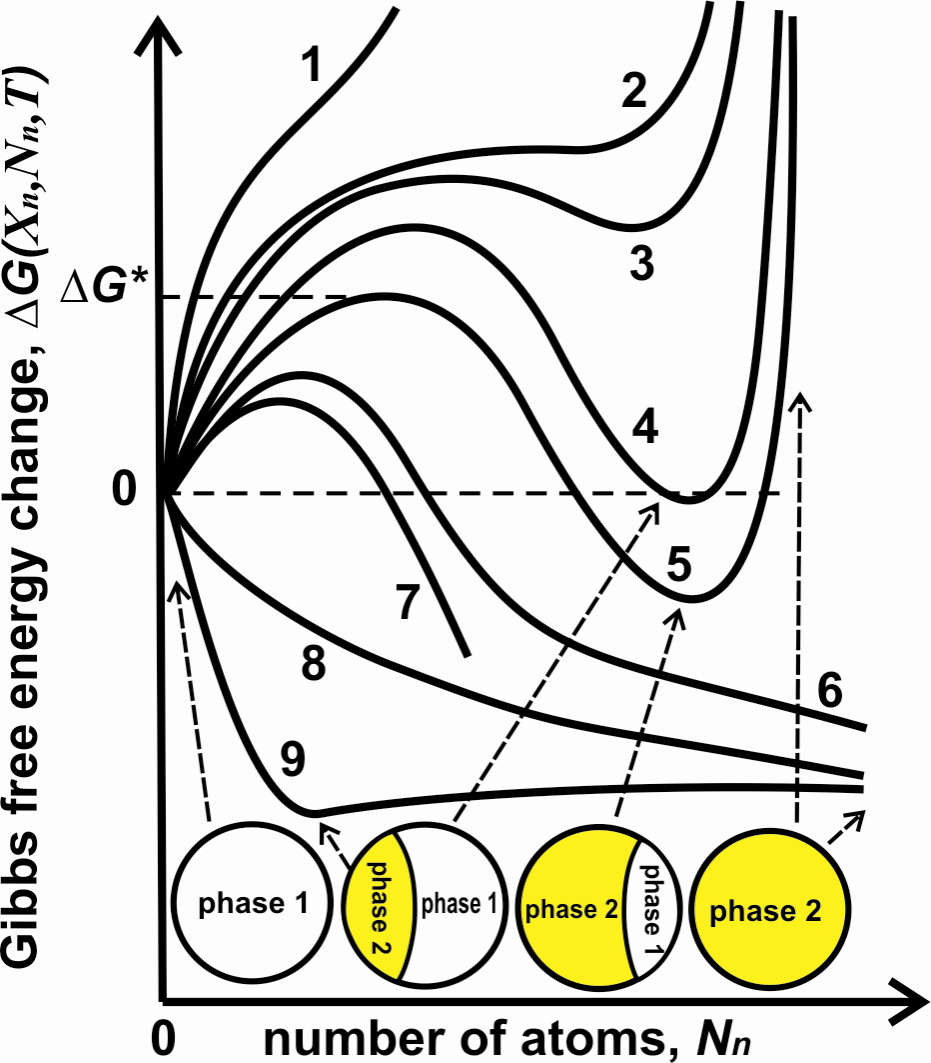
The Gibbs free energy changes Δ*G*(*X*_n_,*N*_n_,*T*) as functions of the number of atoms *N*_n_. The case 7 represents the classical Gibbs thermodynamics and curves 1–6, 8 and 9 – the modification of the classical thermodynamics for transforming multicomponent isolated nanosystems, cases 4, 5 and 9 give stable two-phase states; cases 4–6 and cases 8 and 9 correspond to transition with and without energy barrier, respectively. The right ends of Δ*G*(*X*_n_,*N*_n_,*T*) curves correspond to the totally transformed nanoparticle with new nanophase 2 (as shown in Figure 2c). Cases 6, 8 and 9 show that the nanophase 2 is energetically advantageous as compared with nanophase 1.

At fixed temperature *T*, composition *X*_0_, particle size *N*_0_ and other parameters the system of Equation 10 and Equation 11 yields the dependence Δ*G*(*X*_n_*,N*_n_*,T*) as the function of number of atoms in a new phase *N*_n_ (which is in spherical cases may be of fractional exponent 4/3 and fourth degree of the nucleus size). The function Δ*G*(*X*_n_,*N*_n_,*T*) can be found and depicted by the direct calculation for reasonable compositions *X*_n_ (with small steps *N*_n_). Doing that one shall obtain the curves shown in Figure 3. The classical Gibbs thermodynamics deals with bulk cases and gives the curve with one maximum (case 7 in Figure 3) whereas the modification of the classical thermodynamics for transforming multicomponent nanosystems yields the monotonic as well as nonmonotonic curves with a maximum and minimum (cases 1–6, 8 and 9 in Figure 3). Changing the temperature *T* of the particle (by other fixed parameters) or changing the number *N*_0_ (by other fixed parameters) or alternatively changing only the composition *X*_0_ (by other fixed parameters) it is possible to achieve all states shown in Figure 3.

In two-phase states (Figure 2b) the nanoparticle energy minimum is reached (curve 4 or curve 9 in Figure 3 when Δ*G*(*X*_n_,*N*_n_,*T*) < 0) and each phase has different composition. From this one has to distinguish: 1) initial composition *X*_0_ functioning at the same time as the limit solubility; 2) new phase composition *X*_n_; 3) composition of depleted phase *X*_p_. If one changes the temperature *T* the curves Δ*G*(*X*_n_,*N*_n_,*T)* changes and the minimum point moves as well (for example, cases 4 and 5 in Figure 3). Following those minima of Δ*G*(*X*_n_,*N*_n_,*T*) function one can find all stable states and plot the equilibrium *T–X* nanophase diagram [19]. The letters are presented in next sections.

### Solubility curves

Let us look first on the initial points of the phase transformation by example of melting of the solid nanoparticle of composition *X*_0_ at low values *T*. If one increases the temperature the melting starts when a liquid part appears. This is the solidus temperature (above which both components are no more miscible). By solidus temperature the energy change Δ*G*(*X*_n_,*N*_n_,*T)* has two minima Δ*G*(*X*_n_,*N*_n_,*T*) = 0: first for starting point *N*_n_ = 0 and the second for new two-phase state with *N*_n_ ≠ 0 (for example, case 4 in Figure 3). In some cases it is possible to obtain the situation 9 in Figure 3 when the transition goes without nucleation barrier and Δ*G*(*X*_n_,*N*_n_,*T*) < 0 is already satisfied at the beginning (say, for solid Cu–Ni nanoparticle shown further there exists the wetting condition when the liquid layer covers the solid core or creates the liquid cap at the surface already at low temperatures). Let us call it the transition criterion. If one plots that composition *X*_0_ at *T–X* diagram then it will be point with coordinates (*T*,*X*_0_). Changing the initial composition *X*_0_ and repeating the thermodynamic analysis (Figure 3) one can find the new transition criterion and new solidus temperature. In other words, for the set of compositions *X*_0_ one will have the set of solidus temperatures. The set of solidus temperatures for different values *X*_0_ gives solidus points (*T*,*X*_0_) generating the solubility curve at the *T–X* diagram. In that interpretation solidus is the solubility curve for a solid nanosystem and is the “curve representing in a temperature–concentration diagram the line connecting the temperatures at which fusion is just started for various compositions of a starting solid phase” [19]. The solidus curve becomes size-dependent and indicates only the start of melting but not the two-phase equilibrium. In the following for convenience we call such size-dependent solidus curve as nanosolidus.

In opposite way if one starts from entire liquid nanoparticle at high temperature *T* and decreases it then solidification appears. The corresponding thermodynamic analysis gives shifted and size-dependent liquidus curve (or nanoliquidus) which is “in a temperature–concentration diagram the curve connecting the temperatures at which freezing is just started for various compositions of a starting liquid phase”. The detailed calculation of the nanosolidus and nanoliquidus for particular case of Cu–Ni nanosystem has been done in one of our previous works [20].

### Size-dependent *T–X* diagram

Let us look now on two-phase stable states during the melting of that binary nanoparticle by fixed initial composition *X*_0_ and number of atoms *N*_0_. The solid-to-liquid transformation starts at nanosolidus and goes to new two-phase equilibrium when two phases (depleted phase 1 with changed composition *X*_p_ and new phase 2 with composition *X*_n_) co-exist (Figure 2b and cases 5 and 9 in Figure 3). If one then slightly increases the temperature *T* of that two-phase nanoparticle then it changes the values *N*_n_, *N*_p_ and *X*_n_, *X*_p_ in accordance with changed equilibrium state. As already discussed above these intermediate two-phase states correspond to minima of Δ*G*(*X*_n_,*N*_n_,*T*) curves and can be shown on *T–X* diagram as equilibrium curves. If one plots equilibrium concentrations *X*_n_, *X*_p_ for any fixed *T*, one will obtain a loop-like splitted path (hereinafter referred to a compositional loop or simply loop).

## Results and Discussion

**Figure 4:**
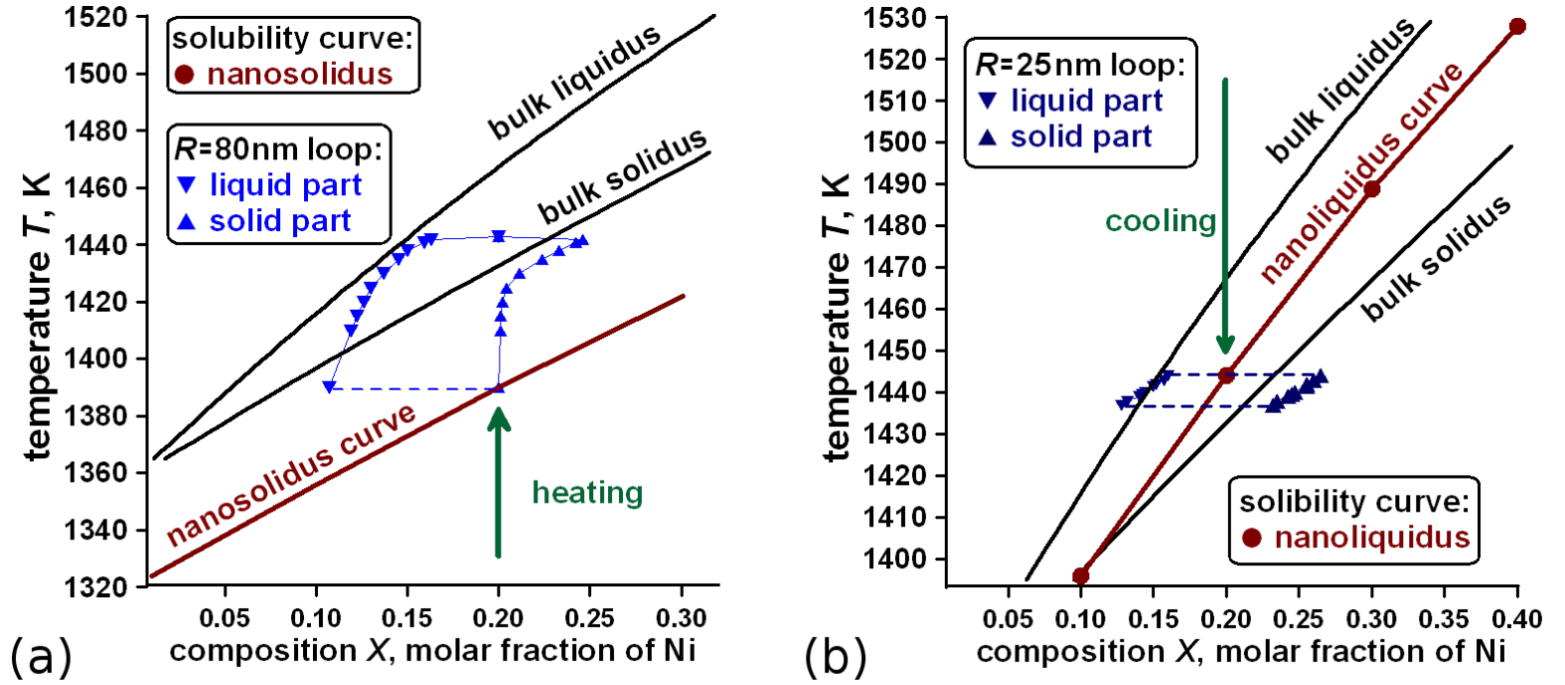
(a) – Nanomelting loop at *T–X* diagram of 80 nm Cu–Ni nanoparticle showing the difference between the compositional loop of two-phase states (two-phase equilibrium states indicated by blue triangle symbols ‘top down’ for liquid part and ‘top up’ for solid part) and solubility curve – nanosolidus (size-dependent solidus curve as starting points of phase transformation indicated by the brown line). (b) – nanosolification loop (indicated by blue triangle symbols ‘top down’ for liquid part and ‘top up’ for solid part) at *T–X* diagram of 25 nm Cu–Ni nanoparticle and solubility curve – nanoliquidus (indicated by brown points and line). Composition *X* is the molar fraction of Ni atoms.

Although the theory is developed in general, we refer to Cu–Ni nanoparticles to highlight major consequences. All thermodynamic values for the Cu–Ni system were taken from the relevant literature [25–40] and given in the Appendix A. Our calculations for individual Cu–Ni nanoparticle show that one must differentiate the solubility curves and the equilibrium loop (Figure 4) discussed here. The former calculations give the solubility *T–X* diagram whereas the letter corresponds to the stability or equilibrium *T–X* phase diagram. In order to see the difference in Figure 4a the result of thermodynamic analysis is presented: size dependent solubility curve – nanosolidus for the radius *R* = 80 nm of the Cu–Ni spherical nanoparticle and one nanomelting loop for initial composition *X*_0_ = 0.2 found by most probable cap-like transformation mechanism (Figure 2b). In Figure 4b we give the corresponding result for *R* = 25 nm Cu–Ni nanoparticle and nanoliquidus found by most probable solid core–liquid shell mechanism of liquid-to-solid transformation. As one can see the solubility curves are not the equilibrium types and say nothing about compositions in two-phase equilibrium states.

**Figure 5:**
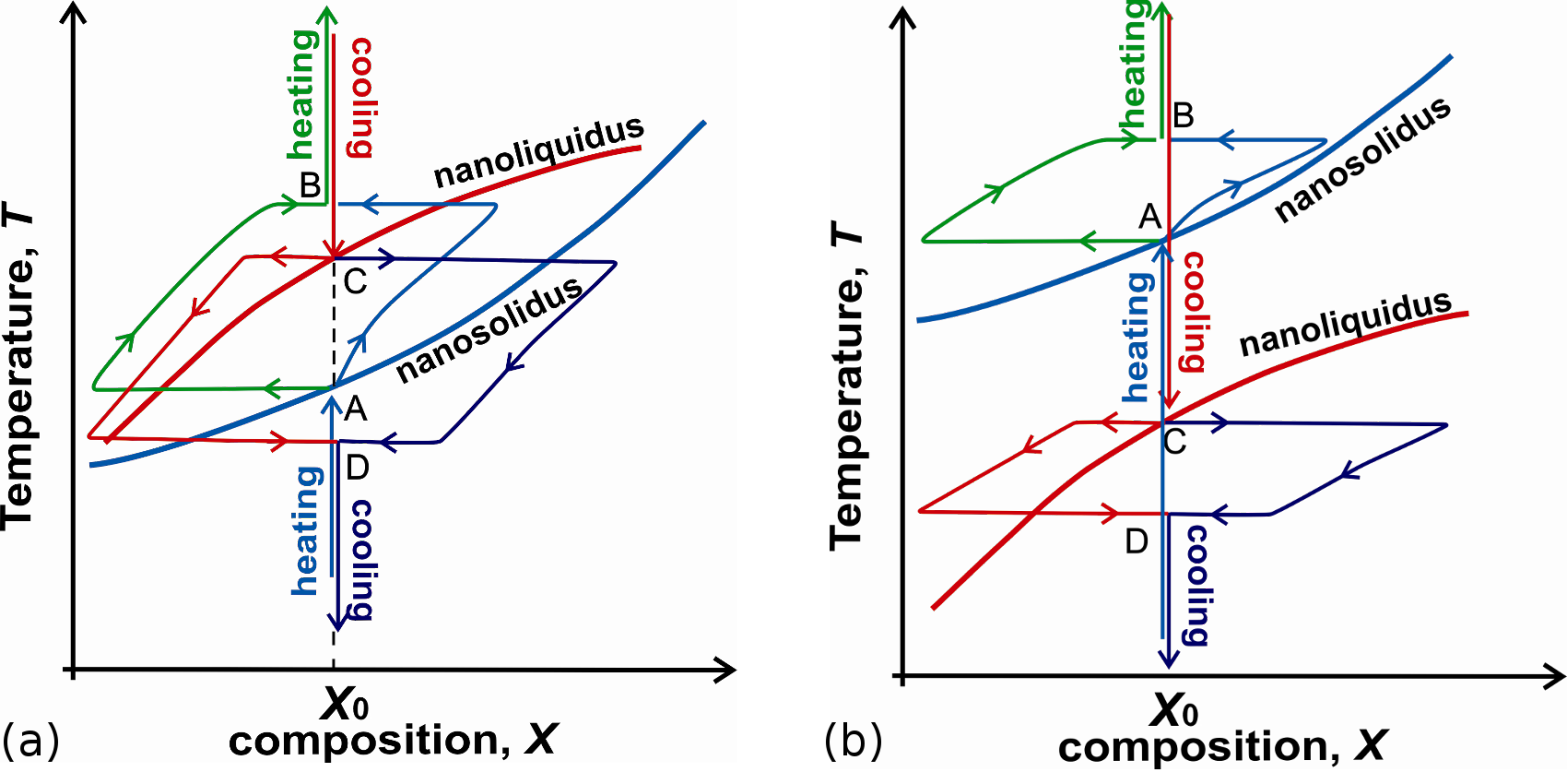
The nanomelting and nanosolidification loops in *T–X* phase diagram in two-phase region for an individual nanoparticle of fixed composition *X*_0_: (a) – the solid-to-liquid loop starts at point A of nanosolidus curve (blue) and ends at point B (green) which is higher than point C for nanoliquidus (red); the liquid-to-solid loop starts at point C of nanoliquidus curve (red) and ends at point D (purple) which is lower than point A for nanosolidus (blue); (b) – nanomelting starts at higher temperatures than the nanosolidification temperatures. The increasing of the sizes leads to the vanishing of the hysteresis effect.

Furthermore, for Cu–Ni nanoparticle we obtained that the solid-to-liquid loop may end at the point which is higher than nanoliquidus. The similar methodology analysis gives that the liquid-to-solid loop starts at point of nanoliquidus curve and ends at point which is lower than the point for nanosolidus. Thus nanosolidus and nanoliquidus may be not interrelated. We call this difference between the end point of forth transition and starting point of back transition as ‘thermodynamic hysteresis’. Similar effect has been shown for a structural transition of Fe-nanoparticle ensemble subjected to temperature change [41]. The reason of such hysteresis is nonsymmetry of transforming path of a nanosystem with respect to the initial conditions leading to differences in two-phase loops of nanomelting and nanosolidification in presented case. For example, for Cu–Ni nanoparticle nonsymmetry is related to the different most probable nucleation mechanisms, namely: forth transition, nanomelting proceeds via cap-like nucleation (Figure 2b) whereas back transition, nanosolification goes through solid core–liquid shell configuration. It means that the forth and back morphologies and the forth and back loops are different (Figure 5) representing the symmetry breaking effect. The increasing of the sizes leads to the vanishing of hysteresis effect.

### Size-dependent solubility diagram

**Figure 6:**
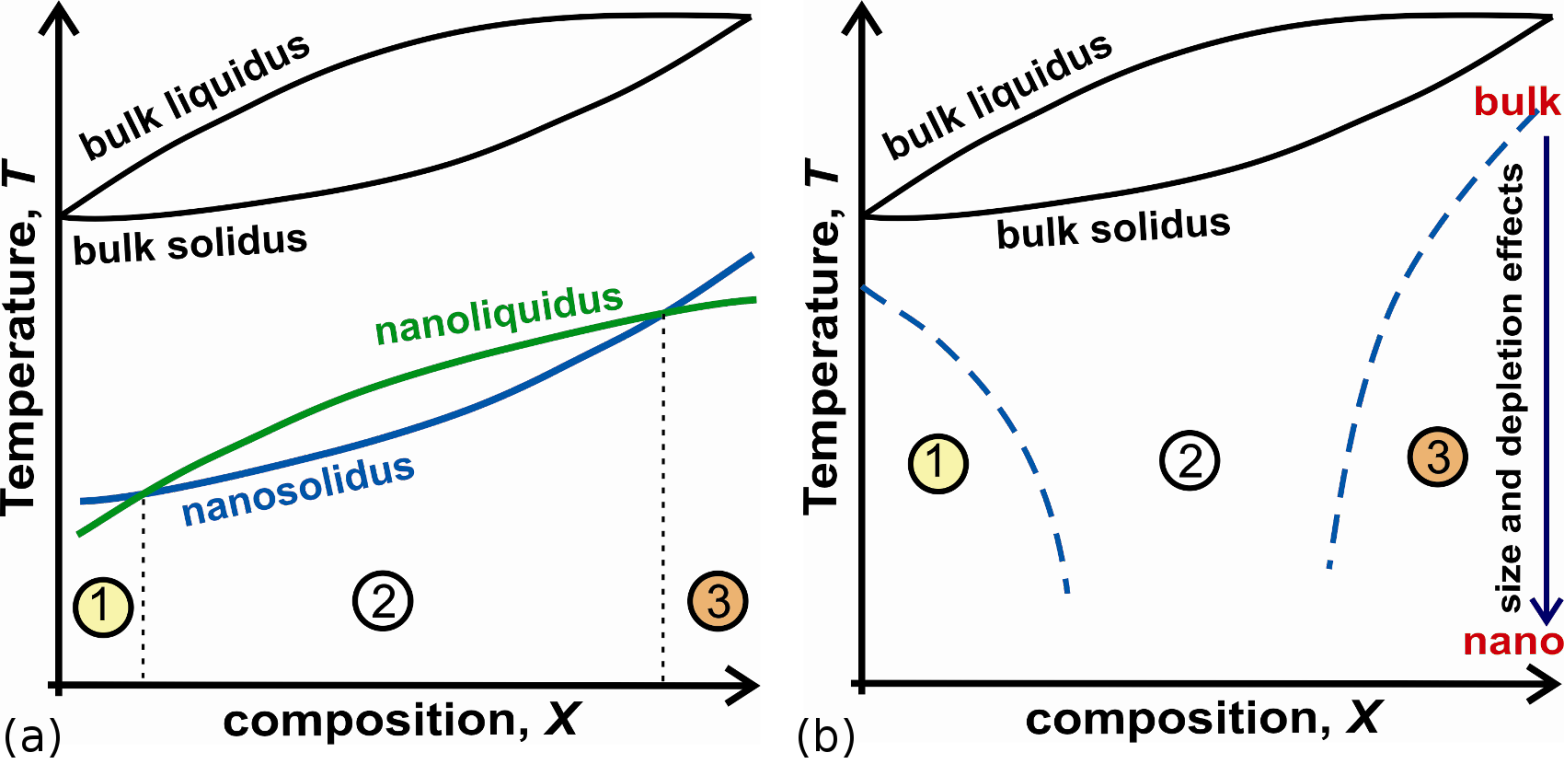
Qualitative representation of size-dependent solubility diagrams for solid–liquid transformation in the isolated Cu–Ni nanoparticle and phase transition symmetry breaking effect shown by the intersection of nanosolidus and nanoliquidus curves and regions (1) and (3) near small and large compositions: (a) – size-dependent shifting and changing of the shape of two-phase region (2) on solubility diagram; (b) – the effect of size on the narrowing of two-phase region (2). The increasing of the sizes leads to increase the width of two-phase region and the vanishing of asymmetry effect (Figure 5).

Next we consider only the solubility curves on *T–X* diagram yielding the size-dependent solubility diagram. Calculations that have been done by us for Cu–Ni and Pb–Bi, Bi–Sn nanosystems [42] at different sizes allow to generalize the picture and resume the size effect on change of the shape and shift of solubility curves and two-phase regions for free nanoparticles and thin films. In general application of the modified Gibbs thermodynamics to free binary nanosystems gives that: i) the solubility curves shift down to lower temperatures, as compared to bulk case, and are the size-dependent; ii) the effective width of the two-phase region on *T–X* diagram decreases as the size of a bimetallic nanoparticle decreases; iii) nanosolidus and nanoliquidus can overlap and intersect; iv) the forms (curvature) of the solubility curves on the diagram change; v) solubility limits, eutectic and peritectic points shift in composition and are size-dependent. In Figure 6 we present the size-dependent solubility diagram for solid–liquid transformation based on the results for Cu–Ni nanoparticles. Hereby the forth and back phase transition symmetry violation is shown as well by the intersection of nanosolidus and nanoliquidus curves and regions (1) and (3) near small and large compositions. The increasing of the sizes leads to the vanishing of asymmetry effect.

## Conclusion

The application of the nucleation and the modification of Gibbs thermodynamics for multicomponent isolated nanosystems are discussed here for the particular case of transforming individual bimetallic nanoparticle. The new-model developments are more general and rigorous than the text-book case which is applicable only for large system sizes where interface contributions can be disregarded.

For the first time, to our knowledge, we calculate and present for individual Cu–Ni nanoparticle the nanomelting loop at size 80 nm and the nanosolidification loop at size 25 nm on temperature–composition phase diagram and generalize the results showing that the nanosolidus and nanoliquidus curves indicate only the starts of nanomelting and nanosolidification but not the two-phase equilibrium. Next new result is that at *T–X* diagram nanomelting loop starts at nanosolidus and ends at temperatures essentially different from nanoliquidus and nanosolidification loop starts at nanoliquidus and ends at temperatures different from nanosolidus. This called thermodynamic hysteresis and it relies on symmetry violation of forth and back transformations in nanosystems. The increasing of the sizes leads to the vanishing of last effect due to decreasing of the surface energy input and vanishing the chemical depletion.

The concept of equilibrium phase diagram has to be revised, due to the fact that the amount of matter is limited in small isolated systems and one needs a new physically acceptable explanation for the purpose of adapting to nanosystems.

## Appendix A: Thermodynamic values for the Cu–Ni system

To calculate the nanomelting and the nanosolidification loops, the Gibbs free energy change Δ*G*(*X*_n_,*N*_n_,*T*) have to be evaluated. A relevant way is using the Gibbs free energy densities *g*_1_(*X*,*T*), *g*_2_(*X*,*T*) of the Cu–Ni system and surface (interphase) energy functions of respective phases σ_1_(*X*,*T*), σ_2_(*X*,*T*) and σ_12_(*X*_1_,*X*_2_,*T*). In our case the temperature interval where liquid-to-solid and vice-versa transitions are investigated is from 1000 to 1700 K. The corresponding thermodynamic data and parameters are used from the literature [25–39] and listed in Table 1.

**Table 1:** The parameters and physico-chemical properties used in calculation of Cu–Ni system [25–39].^a^

Quantity / Property, measure	Cu	Ni

Structure	fcc	fcc
Atomic mass, kg·mol^−1^	*M*(Cu) = 63.55·10^−3^	*M*(Ni) = 58.71·10^−3^
Atomic radii, m	117·10^−12^	115·10^−12^
Bulk melting temperature, K	1357	1728
Bulk boiling temperature, K	2813	within interval 2730–2915
Average atomic volume of solid, m^3^	1.181·10^−29^	1.10·10^−29^
Average atomic volume of liquid, m^3^	1.362·10^−29^	1.253·10^−29^
Average mass density of solid, kg·m^−3^	8950	8910
Temperature dependence of mass density of solid, kg·m^−3^	ρ^S^_Cu_(*T*) *=* 8930 *–* [0.446 + 0.893·10^−4^(*T –* 298.15)] (*T* – 298.15)	ρ^S^_Ni_(*T*) *=* 8900 *–* 0.463·(*T –*298.15)
Temperature dependence of mass density of liquid, kg·m^−3^	ρ^L^_Cu_(*T*) = 7960 *–* 0.76·(*T* – 1357*)*	ρ^L^_Ni_(*T*) = 7850 *–* 1.2·(*T* – 1728)
Relative volume change during the melting, %	4.2	4.6
Average atomic density of solid, m^−3^	8.482·10^28^	9.132·10^28^
Temperature dependence of atomic density of solid, m^−3^	*n*^S^_Cu_(*T*) *= M*(Cu) *N*_A_/ρ^S^_Cu_(*T*)	*n*^S^_Ni_(*T*) *= M*(Ni)·*N*_A_/ρ^S^_Ni_(*T*)
Average atomic density of liquid, m^−3^	7.344·10^28^	7.981·10^28^
Temperature dependence of atomic density of liquid, m^−3^	*n*^L^_Cu_(*T*) *= M*(Cu)·*N*_A_/ρ^L^_Ni_(*T*)	*n*^L^_Ni_(*T*) *= M*(Ni)·*N*_A_/ρ^L^_Ni_(*T*)
Average surface energy of solid, J·m^−2^	σ^S^_Cu_ = 1.731	σ^S^_Ni_ = 2.243
Temperature dependence of surface tension of liquid, J·m^−2^	σ^L^_Cu_(*T*) = 1.321 – 2.260·10^−4^ (*T* – 1357)	σ*^L^*_Ni_(*T*) = 1.810 – 3.925·10^−4^ (*T* – 1728)
Average solid–liquid interface energy, J·m^−2^	σ^SL^_Cu_ = 0.185	σ^SL^_Ni_ = 0.255
Size or radius of nanoparticle, nm	*R =* 25 and *R =* 80	
Number of atoms	*N*_0_ = 5.7·10^6^ for 25 nm and *N*_0_ = 1.867·10^8^ for 80 nm	
Initial composition for nanomelting and nanosolidification, atomic fraction	*X*_0_ = 0.2	
Temperature interval, K	1000–1700	

^a^*N*_A_ is the Avogadro constant, the indexes S and L refer to the solid and liquid and the indexes Cu and Ni refer to chemical elements, respectively.

The calculation of the Gibbs free energy densities of the Cu–Ni binary system is based on the thermodynamic data of the CALPHAD for subregular solutions (taken directly from [40]) and presented in Table 2.

**Table 2:** Thermodynamic data and the Gibbs free energy densities of the liquid and solid Cu–Ni system.

The Gibbs free energy density of the solid Cu–Ni alloy *g_S_*(*X*, *T*), J·mol^−1^

*g*_S_(*X*,*T*) = *X·E*^S^_mix_(Ni) + (*1* − *X*)*·E*^S^_mix_(Cu) + 8.31*·T*·[*X*·ln(*X*) + (1 − *X*)·ln(1 − *X)*] + *X*(1 − *X*)·[*L*^S^_0_ − (2*X* − 1)*L*^S^_1_];
*E*^S^_mix_(Cu) = −7770.458 + 130.485235*·T* − 24.112392*·T·*ln(*T*) − 0.00265684*·T*^2^ + 1.29223*·*10^−7^*·T*^3^ + 52478*·T*^−1^;
*E*^S^_mix_(Ni) = −5179.159 + 117.854*·T* − 22.096*·T* ln(*T*) − 0.0048407*·T*^2^;
*L*^S^_0_ = 8047.7 + 3.42217*·T*;
*L*^S^_1_ = 2041.30 + 0.99714*·T*.
The Gibbs free energy density of the liquid Cu–Ni system *g*_L_(*X*,*T*), J·mol^−1^

*g*_L_(*X*_p_,*T*) = *X·E*^L^_mix_(Ni) + (1 − *X)·E*^L^_mix_(Cu*)* + 8.31*·T·*[(1 − *X)*·ln(*X*) + (1 − *X*)·ln(1 − *X)*] + *X*(1 − *X*)·[*L*^L^_0_ − (2*X* − 1)*L*^L^_1_];
*E*^L^_mix_(Ni) = 11235.527 + 108.457*·T* − 22.096*·T·*ln(T) − 0.0048407*·T^2^* − 3.82318·10^−21^*·T*^7^;
*E*^L^_mix_(Cu) = 12964.84 − 9.510243*·T* − 5.83932·10^−21^*·T*^7^ + *E*^S^_mix_(Cu);
*L*^L^_0_ = 12048.61 + 1.29093*·T*;
*L*^L^_1_ = −1861.61 + 0.94201*·T*.
The expressions for the specific surface energies of the phases, J·m^−2^

σ_S_(*X*,*T*) *= X·*σ^S^_Ni_ + (1 − *X*)·σ^S^_Cu_;
σ_L_(*X*,*T*) *= X·*σ^L^_Ni_(*T*) + (1 − *X*)·σ^L^_Cu_(*T*);
σ_SL_(*X*_n_,*X*_p_,*T*) *= X*_m_*·*σ^SL^_Ni_ + (1 − *X*_m_)·σ^SL^_Cu_, *X*_m_ = (*X*_n_ + *X*_p_)/2.
